# A neural perspective on the treatment of hypertension: the neurological network excitation and inhibition (E/I) imbalance in hypertension

**DOI:** 10.3389/fcvm.2024.1436059

**Published:** 2024-09-11

**Authors:** Min Xia, Tianyu Wang, Yizhu Wang, Tingting Hu, Defang Chen, Bin Wang

**Affiliations:** ^1^ Department of Anesthesiology, General Hospital of The Yangtze River Shipping, Wuhan Brain Hospital, Wuhan, China; ^2^ Liaoning Provincial Key Laboratory of Cerebral Diseases, Department of Physiology, College of Basic Medical Sciences, National-Local Joint Engineering Research Center for Drug Research and Development (R&D) of Neurodegenerative Diseases, Dalian Medical University, Dalian, China; ^3^ College of Pharmacy, Dalian Medical University, Dalian, China; ^4^ Emergency Intensive Care Unit, Qingpu Branch of Zhongshan Hospital, Fudan University, Shanghai, China

**Keywords:** hypertension, neural network, E/I imbalance, neural connection, sympathetic outflow

## Abstract

Despite the increasing number of anti-hypertensive drugs have been developed and used in the clinical setting, persistent deficiencies persist, including issues such as lifelong dosage, combination therapy. Notwithstanding receiving the treatment under enduring these deficiencies, approximately 4 in 5 patients still fail to achieve reliable blood pressure (BP) control. The application of neuromodulation in the context of hypertension presents a pioneering strategy for addressing this condition, con-currently implying a potential central nervous mechanism underlying hypertension onset. We hypothesize that neurological networks, an essential component of maintaining appropriate neurological function, are involved in hypertension. Drawing on both peer-reviewed research and our laboratory investigations, we endeavor to investigate the underlying neural mechanisms involved in hypertension by identifying a close relationship between its onset of hypertension and an excitation and inhibition (E/I) imbalance. In addition to the involvement of excitatory glutamatergic and GABAergic inhibitory system, the pathogenesis of hypertension is also associated with Voltage-gated sodium channels (VGSCs, Nav)-mediated E/I balance. The overloading of glutamate or enhancement of glutamate receptors may be attributed to the E/I imbalance, ultimately triggering hypertension. GABA loss and GABA receptor dysfunction have also proven to be involved. Furthermore, we have identified that abnormalities in sodium channel expression and function alter neural excitability, thereby disturbing E/I balance and potentially serving as a mechanism underlying hypertension. These insights are expected to furnish potential strategies for the advancement of innovative anti-hypertensive therapies and a meaningful reference for the exploration of central nervous system (CNS) targets of anti-hypertensives.

## 1 Introduction

Hypertension has caused a huge burden in mortality and healthcare worldwide due to its huge contribution to the pathogenesis of multiple cardiovascular diseases, such as coronary heart disease, stroke, aortic dissection, and renal events (1, 2). Billions of adults worldwide suffer from hypertension when using a threshold of systolic blood pressure (SBP) ≥140 mmHg and/or diastolic blood pressure (DBP) ≥90 mmHg (3). The magnitude of issue escalates significantly with the adoption of the new threshold of SBP ≥130 mmHg and/or DBP ≥80 mmHg (4). The staggering toll of up to 10.4 million deaths attributed to hypertension in 2017 (5), underscores the imperative for timely assessment and implementation of antihypertensive interventions globally. The majority of hypertension (≥90%) is essential hypertension, of which the pathogenesis is unclear. The central modulation of heart rate (HR) and BP offers a foundation for understanding neurogenic mechanisms underlying hypertension. Besides lifestyle improvements including exercise, reduced alcohol consumption, quitting smoking, and lowering sodium and saturated fats intake could be efficient in controlling hypertension (6), guidelines also recommend the use of diuretics, Angiotensin-Converting Enzyme Inhibitors (ACE/Is), angiotensin II receptor blockers, and calcium channel antagonists, etc. (7). Nonetheless, only about one-fifth of hypertensive patients live with controlled BP (8). Some hypertension that proved refractory escapes from various antihypertensive drugs, which can be effectively managed using deep brain stimulation according to N K Patel et al.'s report (9). This further confirms the potential involvement of a central nervous mechanism in hypertension pathogenesis. With deeper studies in neurology, scientists have found that neural function is not solely reliant on individual neurons but rather on complex neural networks composed of neuronal clusters (10). The dynamic balance of excitation and inhibition (E/I) is necessary for ensuring the optimal functionality of neural networks (11, 12).

Here we review the role of network E/I imbalance in the pathogenesis of hypertension and its potential mechanisms, to improve the understanding of neural mechanisms and narrow the gap in this knowledge. Based on the published research of peers and our lab, the possible neural mechanisms are explored for different etiopathogenesis of hypertension. We focus on hypertension that has received widespread attention over recent years including, but no limited to stress-induced, salt-induced, Angiotensin (Ang) II-induced, and neurogenic hypertension. First, we address the brain regions underlying the genesis of hypertension, and then explore the impact of alternations in these regions at the whole-brain level, such as neural network and neural connection. Accordingly, we have attempted to provide researchers with a new perspective on the pathogenesis of hypertension and with a view to providing some insights into curing hypertension.

## 2 The involvement of the network in hypertension

Previous studies have identified that prenatal malnutrition is tied to an increased risk of hypertension (13, 14). Offspring born to mothers exposed to Dutch famine during pregnancy exhibited heightened vulnerability to hypertension in middle age (15). It may be attributed to the fact that undernutrition during fetal life leads to lower body weight and brain changes, including neuronal loss, impaired neuronal differentiation even neuroplasticity deficits (16), all contributing to the functional aberrations observed in neural networks. Various studies establish that BP regulation by the CNS relies on the coordinated activity of a highly interconnected network of neurons (17, 18). Cayupe et al. reviewed the unfavorable Paraventricular-Coerulear Network arising in the genesis of hypertension in prenatally malnourished adult animals (19). Similarly, hypertension attributed to affective disorders involves neural networks. Hypertension is well-known to be influenced by genetic and environmental factors (20), and so is anxiety formation, especially generalized anxiety disorder (21). A significant anxiety-hypertension association in cross-sectional and prospective studies was pointed out from 59 studies by pooling data recently, even though the underlying pathway is still elusive (22). The pandemic of COVID-19 gave rise to half of the UK adults increased anxiety, depression, or stress due to fear of contracting (23). This heightened anxiety had been associated with a marked increase in cardiovascular events especially hypertension (24). A functional neuroimaging study of anxiety in humans hints at the frequent observation of amygdala and insular cortex (lc) hyperactivation in anxiety disorder (25), change of neural unit in both of which lead to neurogenic hypertension in animals (26, 27). Further animal experiments had made efforts to reveal the mechanism by which the alternation in the brain causes hypertension. They found occlusion of the right middle cerebral artery induces neurochemical disturbances in the amygdala and lc (28), thereby increasing sympathetic outflow and contributing to hypertension in mice (26). Sympathetic overflow increases myocardial contractility and thus stoke volume. Moreover, it also causes vasoconstriction leading increased vascular resistance via the effect of norepinephrine on postsynaptic α-adrenergic receptors of sympathetic nerves innervating the peripheral vasculature. Sympathetic blockade in hypertensive mice decreases cardiac output and peripheral vascular resistance, hence ameliorating hypertension (29). Another research in the context of menopausal women revealed lower intrinsic resting-state functional brain connectivity of the midbrain-brainstem-cerebellar network with bilateral anterior middle insula in hypertension of this population (30).

Sympathovagal imbalance in the central autonomic network may be involved in the pathophysiology of hypertension, given recent evidence confirming the autonomic regulation of the cardiovascular system, which encompasses the insular cortex and anterior cingulate (31, 32). The dense neural projections connect relevant brain regions such as the insula to the autonomic nucleus (33), known for their role in cardiac autonomic regulation (34, 35), offer insights into the underlying mechanism by which these neural connections cause cardiovascular events. A previous animal study confirmed that a high-fat diet leads to changes in neuropeptide gene expression within the CNS, possibly contributing to obesity-induced hypertension and autonomic dysregulation (36). While not directly establishing a causal relationship between autonomic network imbalance and hypertension, this evidence gives few hints. Shiina and his colleagues figured out that improving sympathovagal imbalance is effective in alleviating obstructive sleep apnea-associated hypertension and other adverse cardiovascular events (37). Naiara A Herrera et al. came to a similar conclusion that exercise ameliorates the autonomic balance of dexamethasone-induced hypertensive mice thereby improving the arterial pressure (38). Another acupuncture-based study found that stimulating bilateral TaiChong points in rats enhances the functional connection between the hypothalamus and brain regions related to BP regulation, such as the entorhinal cortex, hippocampal dentate gyrus, brain stem, etc., accompanied by anti-hypertensive effects (39). All these clinical studies corroborate that interventions targeting autonomic network balance alleviate hypertension, confirming to an extent the role of the autonomic network in hypertension pathogenesis. A recent animal experiment proved that autonomic network dysfunction causes hypertension which would be relieved when the autonomic network dysfunction is rescued (40).

## 3 Excitatory glutamatergic system in hypertension

Stress normally produces a temporary rise in arterial BP, with untreated chronic stress triggering persistent hypertension, especially in individuals with a genetic risk for hypertension (41). As the major excitatory neurotransmitter in the vertebrate nervous system, glutamate, along with the excitatory glutamatergic system it comprises, accounts for over 90% of the excitatory synaptic connections in the human brain (42). Glutamate makes its way to the synaptic cleft and acts on downstream receptors and ion channels, either synthesized *de novo* in glutamatergic neurons or recycled through the glutamate-glutamine cycle before being released into the synaptic cleft (43). Excess extracellular glutamate causes neuroexcitotoxicity resulting in an excitatory-inhibitory imbalance in the brain network, thereby precipitating pathological processes (44). The imbalance of the neural network induces excessive sympathetic outflow to trigger hypertension. Given the essential role of the in maintaining neural network homeostasis, its contribution to hypertension warrants investigation.

### 3.1 Stress-induced hypertension

Evidence suggests an important role for chronic stress in the pathogenesis of hypertension, cardiovascular disease, and stroke (45). Our recent publication confirms that chronic stress-induced activation of neurons in the dorsomedial prefrontal cortex (dmPFC) leads to increased glutamate concentrations in the hippocampal vCA1 projection areas of these neurons and elevated BP in mice (46). While not assessing alternations in neural networks excitability, our study observed a rise in glutamate, the main excitatory neurotransmitter, without significant changes in GABA. These results may also hint at the imbalance of E/I in neural networks (42). Basting et al. assessed the effects of acute stimulation of excitatory neurons in the paraventricular nucleus (PVN) selectively on resting BP in awake mice and found it was sufficient to drive an elevation of BP, akin to hypertension (47). PVN integrates the effects of endocrine and autonomic response to stress and ultimately causes hypertension by projecting to sympathetic-related areas in the brainstem and spinal cord leading to a sympathetic overflow (48, 49). A study published recently showed that chronic stress increases sympathetic outflow and ultimately induces hypertension through activation of glutamatergic receptors in PVN and increased binding to the corresponding ligands (41). Overactivation of glutamatergic receptors in PVN is a major source of elevated sympathetic outflow in hypertension (49). Ma et al. have further confirmed that excessive glutamatergic excitatory inputs are strongly linked to the pathogenesis of hypertension (50). Other studies delved into the mechanism of increased glutamatergic tone and found that it was maintained by presynaptic glutamate release and enhanced postsynaptic NMDAR activity (51, 52). Alternatively, research has suggested the upstream glutamatergic output that is projected to the PVN, leading to the activation of the vasopressin (VAP) neurons in it and raising BP (53).

Beyond the PVN, the glutamatergic tonic input of the rostral ventrolateral medulla (RVLM) also results in hypertension (54), as RVLM is a critical site central control of sympathetic outflow playing an essential role in maintaining basal BP (55). Neurons in the RVLM are proposed to send downward impulses to sympathetic preganglionic neurons in the intermediolateral column of the spinal cord (56). Additional glutamatergic input to the RVLM facilitates BP and sympathetic nervous activity (57). ROS-SAPK/JNK signaling pathway regulates the pressor effect of glutamatergic neurons in RVLM in stress-induced hypertensive mice (58). A study modeling stress-induced hypertension based on foot shock combined with noise exposure similarly confirmed the correlation between alterations in the glutamatergic system of RVLM and hypertension. The authors suggested that angiotensin (Ang) II-mediated glutamatergic effects as well as the upregulation of NMDA could activate glutamatergic neurons in the RVLM, releasing glutamate into the intermediolateral column (IML) and altering sympathetic outflow in stress-induced hypertension (59).

### 3.2 Salt-induced hypertension

Numerous epidemiologic, clinical, and experimental studies have confirmed that salt intake is associated with BP and that reducing dietary salt intake could lower BP (60). Normally, salt is often referred to as sodium salt, even though salt constituting only 40% of its composition. Salt-induced increases in BP are in part believed to be mediated by enhanced NaCl in cerebrospinal fluid (CSF) and sympathetic nerve activity (61, 62). Central hypernatremia initiates hypothalamic pathways increasing glutamatergic drive to RVLM neurons, thereby increasing arterial BP (63). An earlier study also found a significant elevation of BP when injecting glutamate into the RVLM of salt-sensitized rats, suggesting a relationship between excessive glutamatergic input and the development of hypertension (64). Changes in gut microbiota are believed to play an important role in salt-induced hypertension. Healthy animals exhibited elevated BP following transplantation of gut bacteria from hypertensive patients or animals. Zheng et al. found that salt overconsumption caused the upregulation of glutamate and its derivatives from comparing the differences between intestinal flora, metabolites, and metabolites pathways in salt-induced rats and controls (65). Subsequent KEGG analysis revealed that the salt-induced alternations were associated with the glutamatergic synaptic pathway, potentially culminating in disruption of E/I balance and consequent hypertension (52). Blocking the angiotensin type 1 receptors (AT1Rs) in the PVN fails to further decrease the peak BP response to the glutamate receptor blockers in salt-induced hypertension while conversely works, probably suggesting the activation of AT1 is primarily mediated by locally released glutamate (66). Also, some research concluded that prohypertensive signals, such as salt can be perceived by autonomic brain regions enhancing resident microglia mainly located in PVN to promote hypertension (67, 68). Instead, attenuation of microglial activation in PVN by administration of minocycline produces antihypertensive effects (69). Stabilized microglia prevent the overactivation of pre-sympathetic neurons in PVN, overactivated microglial constitutive releases platelet-derived growth factor B promoting neuronal potassium current conduction, which in turn triggers excess sympathetic outflow disrupting the excitatory balance of the autonomic system (70). In addition, some researchers stated that brain-derived neurotrophic factor from microglia increases the expression of NMDARs at postsynaptic terminals (71), which might underlie the association of microglial activation with the pathogenesis of hypertension.

### 3.3 Neurogenic hypertension

Sympathetic strain serves as a major pathway in the development of neurogenic hypertension. Basting et al. confirmed the critical role of glutamatergic neurons of PVN in the development of neurogenic hypertension in conscious mice using optogenetics (47). They found a frequency dependent increase in BP upon direct photoactivation of these PVN neurons. Whereas the model of deoxycorticosterone acetate-induced neurogenic hypertension exhibited near-normal BP when these PVN neurons were impaired. The use of calcineurin inhibitors in constructing neurogenic hypertension models has been employed in several studies (72, 73). Elevated synaptic NMDAR activity in pre-sympathetic neurons increased excitability inputs to the PVN, sustaining excessive sympathetic outflow, which may contribute to the genesis of neurogenic hypertension (72). This was supported by an earlier study that NMDAR activation normalize the higher frequency and amplitude of excitatory postsynaptic currents in PVN neurons, which elicits sympathetic tension, a crucial factor in the development of neurogenic hypertension (74). Besides NMDAR, also AMPA-type glutamate receptors (AMPARs) in the postsynaptic membrane, was shown involved in the occurrence of neurogenic hypertension (73). Calcineurin inhibitors initiated an altered phenotype of AMPARs in PVN pre-sympathetic neurons, resulting in an increased Ca2+-permeable AMPARs. These changes triggered an increased postsynaptic currents mediated by AMPAR, leading to excessive sympathetic outflow and the induction of neurogenic hypertension (73). AMPARs are homomeric or heteromeric tetramer consisting of a combination of four pore-forming subunits, GluA1, GluA2, GluA3, and GluA4. A study from Zhou et al. also found that increased synaptic Ca^2+^-permeable AMPARs in PVN presympathetic neuron maintain the sympathetic outflow in neurogenic hypertension by inhibiting the assembly of GluA1/GluA2 heteromers (75). In spite of being glutamate receptors, metabotropic glutamate receptors (mGluRs) do not play exactly the same role in hypertension as the above two. The mGluRs in PVN affect hypothalamic presympathetic neurons via opposing presynaptic and postsynaptic actions (76). Activation of presynaptic group III mGluRs depresses the excitability of PVN presympathetic neurons, attenuating sympathetic vasomotor activity. Conversely, postsynaptic group III mGluRs excite PVN presympathetic neurons, increasing sympathetic vasomotor activity (76).

### 3.4 Ang II-induced hypertension and other hypertension

As a critical site in the development of hypertension, PVN is also identified in other hypertensive models (52, 77, 78). Increased mineralocorticoid receptor leads to enhanced AT1 and glutamate receptor-dependent signaling in the PVN, thereby promoting a chronic increase in circulating Ang II to maintain BP in Ang II-dependent hypertension (77). Another study equally found that enhanced glutamate signaling of PVN, for instance, NMDA-mediated elevation of excitatory currents was present in Ang II-dependent hypertension (78). Central Ang II facilitated glutamate release, that enhanced glutamatergic neuron activation in the RVLM via AT1R binding to enhanced NMDAR and AMPAR, as well as increased sympathetic excitation, causing hypertension in mice (59). Glutamatergic connection activation between the nucleus tract solitarius and the ventral-lateral segment of the medulla oblongata may be one of the baroreflex pathways that produce hypertension (79). Qiao et al. demonstrated that presynaptic and postsynaptic NMDAR activity of RVLM-projecting PVN neurons in essential hypertensive rats whereas blocking this increase lowered BP (52). Nicotine possibly accelerates and deteriorates hypertension by enhancing glutamatergic transmission in spontaneously hypertensive rats (SHR) (80). The glutamatergic signaling network is as well involved in obesity-related hypertension, as the elevated BP induced by excessive central leptin was blocked by glutamatergic inhibition in mice fed a high-fat diet (81). Even blocking the glutamate receptor in postnatal period rescued some of the leptin-induced hypertension from regular feeding (82). All these findings above prompt the pivotal role of the glutamatergic system in the pathophysiology of hypertension and suggests its potential as a reliable strategy for the treatment of essential hypertension.

## 4 Inhibitory GABAergic system in hypertension

The GABAergic (gamma-aminobutyric acid) system serves as the major inhibitory system in the brain, working along with the excitatory system to maintain the delicate balance in the brain for adequate neurological function. Despite making up only 20% of the neurons, GABAergic neurons are vital in generating inhibitory inputs over others (83). As the main bearers of GABAergic signaling, GABAergic receptors (GABARs) were involved in numerous physiopathological processes such as pain, emotion, and cognition (84). As we reviewed above, E/I balance is thought to be implicated in the pathogenesis of hypertension. And what role does the GABAergic system, as an important component of E/I balance, play in achieving hypertension?

### 4.1 Stress-induced hypertension

Over the past decades, it has been well established that stress induces cardiovascular problems such as hypertension, and the regulation of various neurogenic pathways bridges the gap between stress and hypertension as well as cardiovascular disease (85). An MRI-based study showed that GABA levels in the hypothalamus of inherited SHR were significantly lower than in controls, accompanied by an elevated in excitability and energetic activity (86). Another study identified a precise brain region in SHR where E/I imbalance occurs, arguing that the decline in GABA and elevation of glutamate in RVLM may be closely related to the pathogenesis of hypertension (87). They examined neurotransmitter concentrations only in homogenates of tissue from RVLM. It would be more convincing to detect the levels of intercellular neurotransmitter which affect E/I balance in the brain network. Xia et al. examined the intercellular neurotransmitters in the RVLM of SHR treated with PVN injection of melatonin. They discovered that melatonin ameliorated hypertension in SHR while increasing intercellular GABA in the RVLM (88). It hints that altered intercellular GABA-mediated E/I balance in the PVN projective site RVLM closely correlates with the pathogenesis and modulation of hypertension.

To elucidate the mechanisms of neurotransmitter imbalance in the RVLM of SHR, numerous studies are underway. As one of the participants in hypertension, Ang II acts on various receptors, such as AT1R and AT2R. Du et al. found activation of the Ang II pathway plays an important role in SHR by reducing the GABA release (89). However, they did not explain how Ang II caused changed GABA release, which was given by Laura Légat and his team in their recent study. Ang II may cause AT2R activation at synapses in GABAergic neurons, thus altering GABA release (90). It has also been noted that GAD67 expression is decreased in hypertensive rats, ultimately leading to increased vesicle-dependent GABA release (91). Despite these intensive studies were conducted, none of them gave direct evidence to confirm their conclusions. This may await advances in experimental techniques or the launch of extensive research.

### 4.2 Salt-induced hypertension

Given that our daily salt intake far exceeds the recommended limit, health hazards especially cardiovascular risks such as hypertension, are increasingly being emphasized (92). Zheng et al. showed that high salt intake not only affected the excitatory glutamatergic system but also led to a downregulation of GABA. The KEGG enrichment analysis suggested that high salt intake may impact the relevant pathways of GABA metabolism (65). Distinctly, some researchers found that suppressing GABAergic excitation inhibits salt-induced hypertension via reducing the output of AVP neurons (93, 94). Given the inhibitory effect of GABA on neurons, it follows that activating the GABAergic system should inhibit the release of AVP from AVP neurons to attenuate hypertension. They believed that when GABAergic inhibition converts to excitation, the baroreflex loses its function in buffering BP and instead contributes to the development of hypertension via promoting AVP release (94). Kim and his colleague revealed that GABA does not consistently function as an inhibitory neurotransmitter in the brain network according to their study (95). These contradictory studies just indicate that it is unclear what the role of the GABAergic system is in the pathogenesis of hypertension, which is exactly the question that later researchers need to answer.

### 4.3 Neurogenic hypertension

Increased sympathetic outflow is considered to be a major contributor in the pathogenesis of hypertension, with both glutamatergic and GABAergic system being involved (72, 74, 96). PVN pre-sympathetic neurons receive tonic and inhibitory inputs, the balance of which determines the excitability of PVN pre-sympathetic neurons (49). The GABAergic current in PVN neurons of SHR was markedly lower compared with controls, a phenomenon that could be reversed by antagonizing GABARs concomitant with an improvement in hypertension (97). In another study using BPH/2J mice as subjects, it was found that maintenance of hypertension may be due to GABA_A_R structure and function alteration, reducing tonic neuronal inhibition of amygdalohypothalamic network and leading to subsequent sympathetic tension (98). This might account for the disrupted function of GABA receptors and GABAergic inputs to PVN that may lead to increased hypertensive sympathetic outflow in the neurogenic hypertension. It has also been noted a disruption of chloride homeostasis in PVN presympathetic neurons reduces GABAergic inhibition and plays a key role in the hyperexcitability of presympathetic neurons and elevated sympathetic vasomotor tone (96). Numerous studies have identified chronic intermittent hypoxia (CIH) contributes to neurogenic hypertension as well. Short term CIH induces a relatively rapid adaptive response that drives the tonic activity of PVN sympathetically regulated neurons and their acute excitability, a possible mechanism by which CIH evokes neurogenic hypertension (99). Farmer et al. further explored the reason behind this uncontrolled excitation, highlighting the possibility of CIH disrupting GABA_A_-mediated inhibition (100). While a lot of research pointed to a regulatory role for GABAergic system in neurogenic hypertension, its promise as therapeutic target has not been fully demonstrated. Even if some studies have confirmed the therapeutic potential of targeting the GABAergic system through specific injections of the drug in mice brains, an unbridgeable gap remains before an actually use of it for the cure of neurogenic hypertension.

### 4.4 Ang II-induced hypertension and other hypertension

The bed nucleus of the stria terminalis (BST) is a region rich in GABAergic neurons known to regulate cardiovascular parameters. Ang II upregulates the E3 ubiquitination ligase that targets it and promotes angiotensin-converting enzyme 2 (ACE2) ubiquitination and degradation. Onset of this ACE2 ubiquitination in BNST GABAergic neurons decreases PVN presympathetic inputs, promoting hypertension (101). In addition, brain Ang II increases mean arterial pressure and sympathetic outflow by working on AT1R in diverse parts of the brain, including the PVN, RVLM and nucleus of the solitary tract (NTS) (90). Ang II mediated stimulation of AT1R in the NTS leads to activation of eNOS or (and) nNOS, followed by local production of NO, enhancing GABA release from NTS interneurons (90). GABA release in the NTS inhibits glutamatergic neuronal projections to the caudal ventrolateral medulla (CVLM), reducing activation of inhibitory GABAergic nerves in CVLM and subsequently deregulating sympathetically-driven glutamatergic neurons in the RVLM (102). Control of GABAergic is also exhibited in other forms of hypertension. Haywood et al. confirmed that injection of GABA receptor inhibition in bilateral PVN resulted in a significant increase in BP and HR in renal-wrap hypertensive rats (103). They also concluded stronger GABAergic activity is the outcome of increased GABA release from the PVN (104). Maycon et al. instead uncovered that peripheral GABAergic control may also be part of the pathogenesis of renal hypertension, suggesting the equal importance of peripheral and central E/I balance in the pathogenesis of hypertension (105). Likewise, exercise could elevate glutamic acid decarboxylase (GAD) expression, which increases GABA levels to attenuate renal sympathoexcitability and hypertension by diminishing GABAergic deficits in the caudal hypothalamus (106, 107).

## 5 Nav isoforms and the potential effects of Nav-mediated E/I in hypertension

Chronic stress has garnered significant attention due to its capacity to trigger a cascade of pathophysiologic alterations, thereby contributing to kinds of cardiovascular disease via the (CNS) (85). For instance, sympathetic overactivity and neuronal excitation imbalance in the brain are involved in the regulation of hypertension (108). Except for chronic stress, sodium (Na^+^) intake is another environmental factor known to influence BP; increased Na^+^ heightens an individual's susceptibility to hypertension while the reduction of Na^+^ intake alleviates hypertensives (109). In human beings, there is a continuum of BP sensitivity to sodium, which is related to vascular endothelium and smooth muscle, renin-angiotensin-aldosterone system, immune responsive system. Additionally, the specific regions of the brain would influence the salt-sensing variability (110–112). It has been reported that epithelial sodium channels contribute to the salt sensitivity of BP in the kidney (113), the organ primarily responsible for sodium homeostasis.

Recent studies have found that function or expression changes of sodium, potassium, and calcium plasma channels in specific brain regions are related to the regulation of hypertension (114). Among these channels, Nav are normally responsible for the generation and propagation of action potentials in neurons, which directly affects the excitability of neurons and is involved in the regulation of Ca^2+^ transfer and the release of glutamate and/or GABA at the end of axons (115). The mammalian Nav family consists of 10 α-subunit genes encoding Nav1.1–Nav1.9. Nav1.1–1.9, each of which is expressed in different tissues and exert different functions. Mutations in Nav1.1 result in altered sodium channel activity occurring primarily on inhibitory interneurons with consequent slow recovery from inactivation, greater use-dependent inactivation and reduced action potential firing in interneurons. These changes ultimately lead to diminished inhibition and E/I imbalance within the brain (116). By contrast, Nav1.2 mutations caused a reduction in Na^+^ currents primarily on pyramidal neurons, with decreased excitatory synaptic inputs to pyramidal neurons in brain slices, whereas interneurons were unaffected, which biased the brain towards an inhibitory imbalance (117). Mice heterozygous of Nav1.6 had neurons in hippocampal CA1 in a state of hyperexcitability with a significant increase in the rate of rising rate of action potentials, spike amplitude and input resistance. Neuronal excitability in the cortex was also increased, which have led to the neural network hyperexcitability. Apart from this, these mice exhibited simultaneous tonic extension and bradycardia on ECG (118). Some other types of Nav introduce E/I imbalance response as well, which we will elaborate on in the following sections. Along with the direct alterations in Nav, the auxiliary protein fibroblast growth factor 13, which encodes Nav, would lead to decreased inhibitory synaptic inputs and increased excitatory synaptic inputs, leading to an E/I imbalance in the neural networks (119). In the CNS, Nav-mediated abnormal electrical activity of neurons can induce the neurological network E/I imbalance and lead to anxiety (120, 121). Then, would the Nav mutations in the brain affect the BP and the formation of hypertension? Our laboratory has long been involved in research related to ion channels, and we hypothesize the existence of additional central mechanisms involvement of ion channels in hypertension.

### 5.1 Stress-induced hypertension

Various Nav isoforms expressed in different tissues and exert different functions. Na1.1, Na1.2, Nav1.3, and Na1.6 are the primary isoforms expressed in the CNS, Nav1.4 is distributed in skeletal muscle, and Nav1.5 is only expressed in cardiac muscle. Especially, Nav1.6 is highly expressed in excitatory neurons, and is also shown in inhibitory neurons. We have reported that downregulating or inhibiting Nav1.6 suppresses intracellular Ca^2+^ accumulation, thereby attenuating the pathogenesis of Alzheimer's disease (122) and 6-OHDA-induced neurotoxicity and neuroinflammation (123). Recent findings by Lei Tong et al. declared that Nav1.6 is overexpressed in the RVLM neurons in SHR, accompanied by increased vesicular glutamate transporter 1 (VGluT1) expression and VGluT1-positive neurons, alongside decreased GAD67 expression and GAD67-labeled inhibitory neurons (124). In stress-induced hypertensive rats subjected to electrical foot and noise, the Nav1.6 expression in RVLM is markedly increased, whereas knockdown the Nav1.6 in RVLM significantly ameliorates stress-induced in SBP, HR and sympathetic nerve activity in rats (125). Calcium ion is the switch for glutamate release, and GAD catalyzes the conversion of glutamate to GABA. These indicate the E/I imbalance, resulting from the overactivated glutamate system and (or) inhibited GABAergic system, may be induced by increased Nav1.6 and Ca^2+^ accumulation, thereby mediating the stress-induced elevation of BP.

No reports are available to reveal a correlation between Nav1.7 and hypertension, although attempts to explore this area have concluded a lack of association (126, 127). Nav1.8 in the brain is mainly expressed in a few regions including lateral septal nucleus, BST, dorsal striatum, amygdala, hypothalamus, and the ventral periaqueductal gray (128), several of which may be involved in the pathogenesis of hypertension (26, 39, 101). The involvement of Nav1.8 in prolactin-regulated stress-induced behavior response was found in a study on preclinical migraine models done by Bianca and his colleagues (129). Another study also confirmed that stress-induced an upregulation of neuronal Nav1.8 expression, although this alteration was transient (130), which was corroborated by Helia et al. on their discovery that neurons transiently expressing Nav1.8 were able to sense noxious stimuli in the brain (128). Upregulated of Nav1.8 in prefrontal neurons undergoes excitatory deficits including reduced spiking frequency of action potential and depolarization of resting membrane potentials (131). Knockout out of Nav1.8 on dorsal root ganglion neurons prevents them from emitting all-or-none action potentials at the resting potential state (132). Above studies confirm the involvement of Nav1.8 on CNS and peripheral neurons trigger deficits in neuronal excitability, but lack evidence tying it to E/I imbalance and hypertension which deserves further exploration.

### 5.2 Salt-induced hypertension

Neuronal activity is affected by the concentration of Na^+^ in the CSF, which is extremely sensitive. An increase of 2 mmol/L in the Na^+^ concentration in the CSF triggers neuronal discharges (133), whereas chronic elevation of CSF [Na^+^] by 5 mmol/L results in sympathetic hyperactivity and hypertension (134). The sodium concentration in this fluid depends on the sodium transport between plasma and CSF by CP epithelial cells through the epithelial sodium channels (ENaCs) and Na ^+ ^-K ^+ ^-ATPase. High-salt diet increased the concentration of sodium ions in the CSF of rats, which led to the increase of sympathetic nervous system excitability, BP and HR, and these effects were more significant in Dahl-sensitive rats (135). The abnormal regulation of Na^+^ transport and Na ^+ ^-K ^+ ^-ATPase activity may be one of the reasons for the increase of [Na^+^] in CSF in Dahl salt-sensitive rats fed a high-salt diet, but not related to the abnormal regulation of ENaCs (136). They also found the reduction of [Na^+^] influx caused by blocking ENaCs by benzamil still resulted in elevated [Na^+^] in CSF even leaving the salt diet (136). It reflects the key role of ENaCs of transporting Na^+^ in salt-induced hypertension. Furthermore, the sympathetic and pressor responses to intracerebroventricular injection of sodium-rich artificial CSF were greater in Dahl S Rats than in salt-insensitive (R) or Wistar rats (135). This may be due to the acute and chronic increases in [Na^+^] that reduce NO release, leading to an imbalance of E/I and increasing Na ^+ ^-induced hypertension via enhancing Ang-II release and AT1R activation in PVN (137).

### 5.3 Neurogenic hypertension and Ang II-induced hypertension

Some debate over the notion that excessive sodium intake leads to elevated [Na^+^] or CSF [Na^+^] which later causes hypertension. Nomura et al., in studying neurogenic hypertension, found that Nax (encoded by SCN7A) knock out mice failed to manifest mean blood pressure increases even after high-salt ingestion (138). Elevated extracellular [Na^+^] in the organum vasculosum lamina terminalis (OVLT) activates Nax, which stimulates the release of H^+^ from Nax-positive glial cells. The released H^+^ provokes OVLT (projecting to PVN) neurons to further activate PVN (projecting to RVLM) neurons, thereby increasing sympathetic activity and leading to hypertension. The OVLT's significant role in neurogenic hypertension likely stems from its lack of a typical blood-brain barrier and proximity to the third ventricle, allowing it to sense increased [Na^+^] in both the blood and CSF (138). This strained sympathetic activity is associated with elevated Nav1.6 expression in the RVLM, and reducing Nav1.6 expression results in decreased sympathetic activity (125). Overexpression of Nav1.6 leads to intracellular calcium accumulation via Na ^+ ^-Ca^2+^ exchange in the brain (123), triggering excessive release of excitatory neurotransmitters thus initiating E/I imbalance (139). However, definitive evidence linking Nav1.6 to neurogenic hypertension through these mechanisms is still lacking, requiring further research. Gabor et al. demonstrate that glutamate receptor-dependent signaling in the PVN contributes to the BP maintenance in Ang II-hypertensive Wistar rats (77). Despite overexpression of Nav1.6 could bring excessive glutamate release based on previous studies (122, 123), it is difficult to justify the role of Nav1.6 in development of Ang II-dependent hypertension. More than just neurogenic hypertension, the role of Nav in the pathogenesis of Ang II-induced hypertension needs more exploration.

## 6 Conclusion

**Figure 1:**
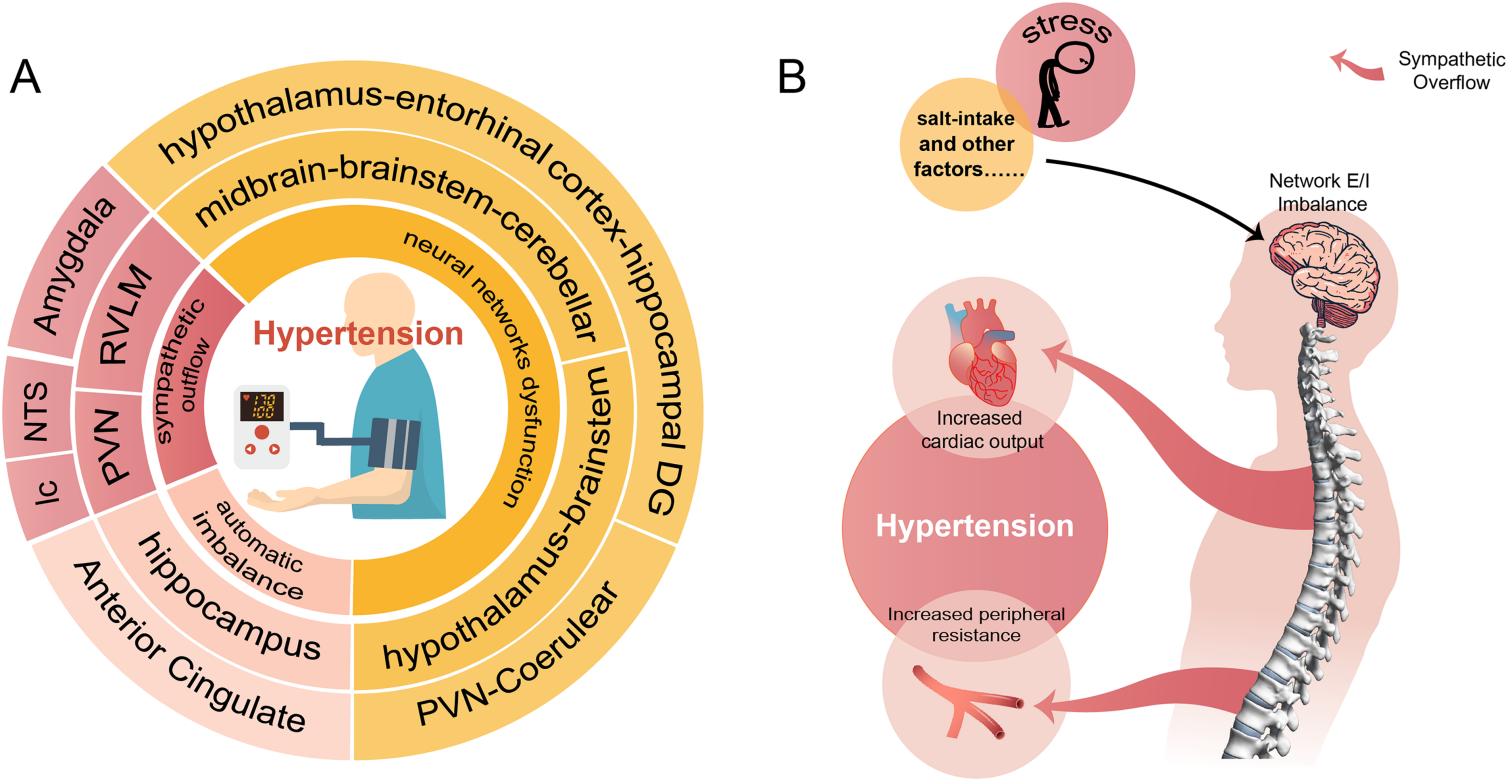
A conclusion of the neural network involved in the pathogenesis of hypertension. **(A)** Shows that hypertension involves neural mechanisms (innermost circle) and corresponding brain regions (two outer circles of the same color). **(B)** Demonstrates the process of external stimuli causing hypertension through disturbed neural networks and sympathetic overflow.

Hypertension is so far incurable that current treatment relying on medications to control the BP. Most hypertensive patients are required lifelong use of two or more antihypertensive drugs, leading to reduced compliance and disruption of normal routines. We reviewed the important role of neural network E/I imbalance in the pathogenesis of hypertension (Figure 1), suggesting its potential as a therapeutic target. This opens the possibility of exploring approaches such as transcutaneous or transcranial electrical stimulation targeting specific brain regions, nano-delivery systems, and targeted drugs to optimize disturbed brain network. These interventions could potentially lead to a curable outcome for hypertension, enabling patients to live a routine life free from lifelong medication.
